# The Importance of Distance to Resources in the Spatial Modelling of Bat Foraging Habitat

**DOI:** 10.1371/journal.pone.0019227

**Published:** 2011-04-25

**Authors:** Ana Rainho, Jorge M. Palmeirim

**Affiliations:** 1 Departamento de Biologia Animal and Centro de Biologia Ambiental, Faculdade de Ciências, Universidade de Lisboa, Lisboa, Portugal; 2 Instituto da Conservação da Natureza e da Biodiversidade, Lisboa, Portugal; University of Western Ontario, Canada

## Abstract

Many bats are threatened by habitat loss, but opportunities to manage their habitats are now increasing. Success of management depends greatly on the capacity to determine where and how interventions should take place, so models predicting how animals use landscapes are important to plan them. Bats are quite distinctive in the way they use space for foraging because (i) most are colonial central-place foragers and (ii) exploit scattered and distant resources, although this increases flying costs. To evaluate how important distances to resources are in modelling foraging bat habitat suitability, we radio-tracked two cave-dwelling species of conservation concern (*Rhinolophus mehelyi* and *Miniopterus schreibersii*) in a Mediterranean landscape. Habitat and distance variables were evaluated using logistic regression modelling. Distance variables greatly increased the performance of models, and distance to roost and to drinking water could alone explain 86 and 73% of the use of space by *M. schreibersii* and *R. mehelyi*, respectively. Land-cover and soil productivity also provided a significant contribution to the final models. Habitat suitability maps generated by models with and without distance variables differed substantially, confirming the shortcomings of maps generated without distance variables. Indeed, areas shown as highly suitable in maps generated without distance variables proved poorly suitable when distance variables were also considered. We concluded that distances to resources are determinant in the way bats forage across the landscape, and that using distance variables substantially improves the accuracy of suitability maps generated with spatially explicit models. Consequently, modelling with these variables is important to guide habitat management in bats and similarly mobile animals, particularly if they are central-place foragers or depend on spatially scarce resources.

## Introduction

Bats are a highly speciose group that usually makes up a major proportion of the mammalian diversity of temperate and tropical ecosystems, but many of its species are increasingly threatened (see [1]). The destruction or degradation of their foraging habitats, usually a consequence of land use intensification, is one of the main factors that is contributing to the decline of bat populations [1]. Even species that can thrive in agro-ecosystems are being affected by changes in agricultural practices that decrease the quality of farmland as foraging habitat [2]. However, in much of the World, especially in Europe and North America, efforts to manage habitats to benefit threatened species, including for those living in agricultural landscapes, are rising. Environmental and agricultural funds are increasingly being used to maintain or even improve the habitat suitability, but success depends greatly on our capacity to determine where and how management interventions should take place [3]. Spatially explicit multivariate models of habitat suitability are becoming a very important tool in the planning of those interventions, and their development is an active area of ecological research (see [4] for a review). When modelling foraging habitat suitability for a species it may be important to include not only the potential quality of the habitats, but also elements of the species' biology that may determine how it uses foraging space, and this topic has received little attention in the case of bats (but see [5], [6]).

Flight allows most bats to travel long distances, which usually gives them access to resources scattered widely in the landscape. The places where bats roost, drink, and forage are often kilometres apart, and the spatial distribution of these resources may be dynamic, as in the case of species that depend on spatially rare and temporary sources of food, such as fruiting trees and insect swarms [7]. As a result, some bat species feed up to a few dozen kilometres away from their colonial roost so their colonies can have foraging ranges covering a few thousand square kilometres (e.g. [8], [9]). Optimal foraging theory predicts that foragers tend to maximise the long-term net rate of energetic gain, and thus have to consider time and energy costs while collecting food (e.g. [10]). Flying is energetically costly, so travelling distance may constrain the choice of foraging areas by bats. In fact, potentially good foraging sites may remain unused simply because reaching them is energetically unsustainable. Consequently, distance variables are potentially important for modelling foraging habitat selection by bats. Examples of distance variables that may influence how bats use foraging space include the distance from potential foraging sites to day roost, to sources of drinking water, and to urban areas.

Colonial species, like most bats and many birds, are considered central-place foragers [11], [12], [13], because they usually return to the same site after foraging [14]. This need to return to the roost forces many bats to make long commuting flights to the foraging areas. *Tadarida teniotis*, for instance, can commute daily to foraging sites located up to 30 km from the day roost [8]. Although such long flights are often done at speeds that minimize energetic expenditure [15], they are inevitably costly and bats presumably only make them to reach particularly rewarding foraging sites. Indeed, in the above referred case of *T. teniotis* most of the foraging areas were within 5 km from the roost [8].

In dry regions, such as the Mediterranean, high ambient temperature combined with low relative humidity causes high rates of evaporative water loss in bats. Under these conditions, bats may lose as much as 30% of their body water over a 12-hour period [16], which must be replenished in part by drinking [17]. At least some bat species drink during their night foraging activity [18], so the proximity to water sources may prove important in the selection of a foraging area in dry regions. In fact, the availability of drinking water is likely to be one of the most general factors influencing the use of space by vertebrates [19], [20], [21].

The number of street lights tends to increase with the proximity of urban areas, and this increase in artificial night lighting is known to be important to bats. In fact, street lights are known to attract some groups of insects, creating spots of high prey abundance that attract foraging bats of several species [22], [23]. For these bat species, urban and suburban street lighting may increase the suitability of foraging habitat. However, there is also evidence that some bat species avoid artificially illuminated areas, presumably to minimize the risks of predation [24], [25], so for these species the densification of street lighting, may lower the suitability for foraging. Many taxa of other vertebrate groups, including birds [26], [27] and amphibians and reptiles [28], [29], are also known to be influenced in their foraging activities by street lightening, which may have an effect on habitat suitability.

It follows from this potential importance of distance variables as determinants of the way bats use the landscape, that they should be included in the evaluation of bat foraging habitat suitability. However, most modelling studies of bat foraging habitat only include land-cover, using it as a surrogate of all relevant environmental variables (e.g. [8], [30]). Useful as it may be, this approach may lead to erroneous conclusions, as it has been demonstrated for some central-place foraging birds [11].

The integration of distance variables in foraging habitat evaluation requires a multivariate modelling approach, where they can be considered simultaneously with land-cover and other potentially relevant landscape variables, and this has been done in a few studies with birds [19], [31]. Distance variables have been used in the modelling of the geographic distribution of bats [32], [33] and one study included them in the modelling of foraging habitat [6]. However, to our knowledge, there are no studies focused on the evaluation and discussion of the potential contribution of distance variables to the accuracy of bat foraging habitat models.

Our main objective was to assess the importance of distance variables as predictors in the modelling of bat foraging habitat in heterogeneous landscapes, using as models two bat species of conservation concern, *Miniopterus schreibersii* and *Rhinolophus mehelyi*. In particular, we evaluated influence of distance to day roost, distance to water, and distance to urban areas, in a Mediterranean agricultural landscape. In addition, we developed spatially explicit multivariate models that integrate distance and habitat variables for both species. Such models allowed the construction of foraging habitat suitability maps, which may help determining the areas where management should be concentrated.

## Results

A total of 31 adult female bats were radio-tracked, and foraging data were successfully recorded for 13 *M. schreibersii* and 12 *R. mehelyi* (see Table S1 for details on tracking data). Bats used the same day roost throughout each tracking season.

### Colony home range

**Figure 1:**
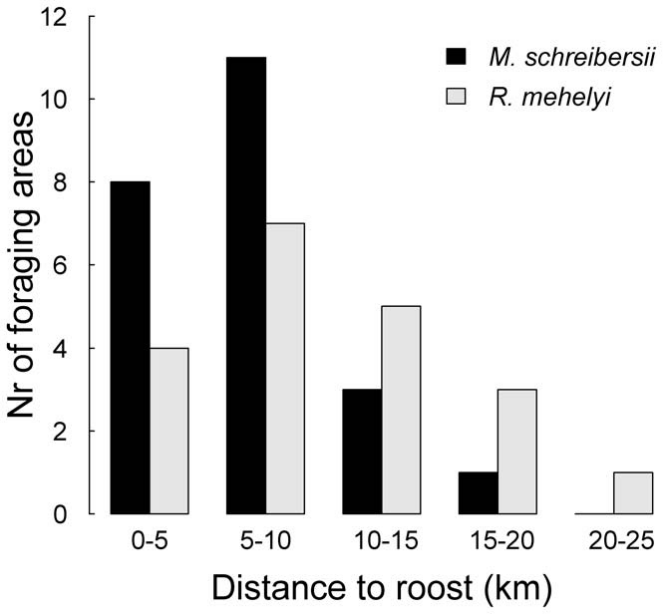
Linear distance between the roost and the centre of each foraging area located during this study. Most of these areas were used on multiple nights by the same bat.

After leaving the roost, bats flew directly to their foraging areas. We mapped 22 such foraging areas for *M. schreibersii* and 20 for *R. mehelyi*. During its tracking period each bat showed high fidelity to one or two neighbouring areas, to which it returned every night. Both species covered large distances to reach appropriate foraging grounds, but while a radius of 10 km included 82% of the foraging areas of *M. schreibersii*, it included only 52% of those of *R. mehelyi* (Figure 1). The foraging areas furthest away from the roost were at 15.5 km for *M. schreibersii* and 22.3 km for *R. mehelyi*.

### Habitat suitability modelling

**Figure 2:**
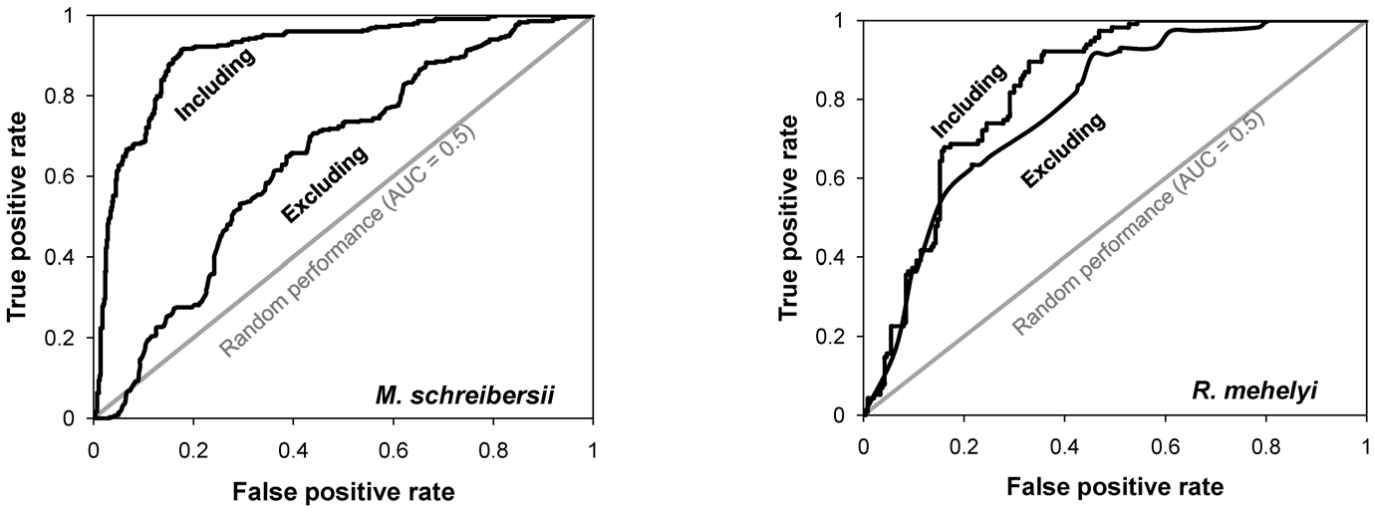
Comparison of the performance between selected models including and excluding distance variables. The comparison was performed using the area under the ROC curve (AUC). Note the low performance (smaller AUC) of the models that exclude the distance variables, particularly for *M. schreibersii* (see Table 2 for full model statistics).

All the models that used the datasets without distance variables performed quite poorly (Table 1). The best of these models for *M. schreibersii* had an area under the receiver operating characteristic (ROC) curve (AUC) of just 0.64±0.01 (*P*<0.0001), and for *R. mehelyi* an AUC of 0.78±0.03 (*P*<0.0001) (Figure 2). In contrast, for both species, some of the models based on the datasets that included distance variables had a high fit and excellent discrimination ability (Table 1). The best model for *M. schreibersii* included, in addition to landscape descriptors, three distance variables (distance to roost, to water, and to light), and had an AUC of 0.91±0.01 (*P*<0.0001). The best model for *R. mehelyi* included soil productivity and two distance variables (distance to roost and to water), and had an AUC of 0.83±0.02 (*P*<0.0001) (Figure 2).

**Table 1:** Best candidate habitat suitability models for the foraging data of *Miniopterus schreibersii* (top) and *Rhinolophus mehelyi* (bottom) based on an information-theoretic approach using the Akaike information criterion for small samples (AICc).

	Models		Unconditional Std Error	Unconditional Conf Intervals	AICc (univariate)
***M. schreibersii***	**1**	**2**			
Deviance	671	1147			
AIC_c_	693	1159			
Δ_i_	0	466			
*w_i_*	1.00	0.00			
Intercept	4.85	0.02	0.77	[3.31–6.34]	
Dist water	0.12		0.31	[−0.69–0.52]	1150
Dist water ^2^	−0.10		0.06	[−0.18–0.08]	
Dist roost	−0.05		0.11	[−0.26–−0.01]	810
Dist roost ^2^	−0.02		0.00	[−0.03–−0.01]	
Dist urban	−0.37		0.06	[−0.49–−0.26]	1092
Altitude	−0.01	−0.01	0.00	[−0.02–0.00]	1198
Landcover					1165
Montado(d)	0.30	−0.36	0.41	[−0.62–0.98]	
Olive grove	1.03	0.78	0.30	[0.30–1.48]	
Open area	0.96	0.94	0.29	[0.42–1.56]	
Other	0.99	−0.12	0.49	[−0.08–1.83]	
***R. mehelyi***	**1**	**2**			
Deviance	329	361			
AIC_c_	337	374			
Δ_i_	0	37			
*w_i_*	1.00	0.00			
Intercept	3.72	0.84	0.75	[1.86–4.79]	
Dist water	−0.35		0.14	[−0.65–−0.11]	429
Dist roost	−0.14		0.03	[−0.20–−0.09]	419
Soil	−0.86	−0.70	0.13	[−1.07–−0.57]	368
Landcover					416
Montado(d)		0.33	0.56	[−0.77–1.44]	
Olive grove		0.63	0.66	[−0.79–1.80]	
Open area		0.49	0.58	[−0.71–1.57]	
Other		0.07	0.55	[−0.75–1.39]	

In model 1 all significant variables were considered, while in model 2, distance variables were excluded. Both models are ranked by AICc differences (Δ*i*). The table indicates for each model: deviance (D2), AICc, Δ*i*, Akaike weights based on the entire set of models (*wi*). The coefficient for each variable is reported together with the AICc for the univariate model, and the unconditional standard error and confidence intervals (not conditional on any particular model) as measures of the precision of coefficients.

**Figure 3:**
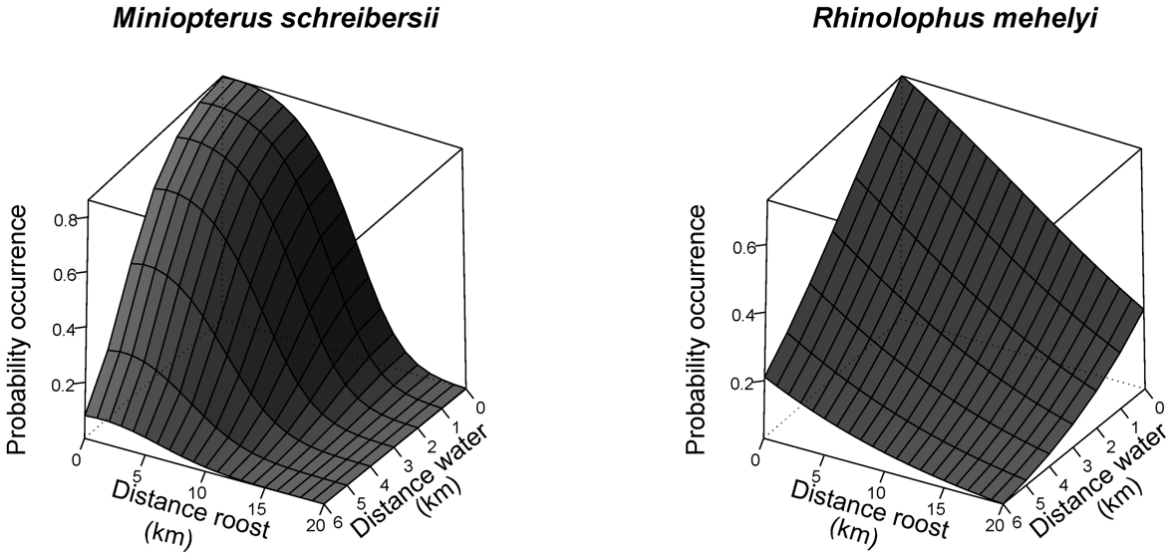
Estimated probability of occurrence of foraging bats, based on models using distance to roost and distance to water. Notice the great power of the two most important distance variables for both species, to explain the probability of occurrence.

The importance of distance variables is such that models using just distance to roost and to water, could explain as much as 86% and 73% of the probability of occurrence of *M. schreibersii* and *R. mehelyi*, respectively (Figure 3). However, the way these variables explain the probability of occurrence of the two species is nevertheless quite distinct. The negative quadratic variation of probability in response to distance to roost indicates that *M. schreibersii* strongly favours areas close to the roost, with suitability declining rapidly beyond a threshold distance of about 5 km (Figure 3). In the case of *R. mehelyi*, suitability declines steadily with distance to roost, and they tend to use distant areas more than *M. schreibersii*. Both species tend to favour the proximity to waterlines, but more markedly so in the case of *M. schreibersii* (Figure 3). The two species responded differently to the distance to public illumination; it increased the foraging habitat suitability for *M. schreibersii* but was irrelevant for *R. mehelyi* (Table 1).

### Predictive habitat suitability maps

**Figure 4:**
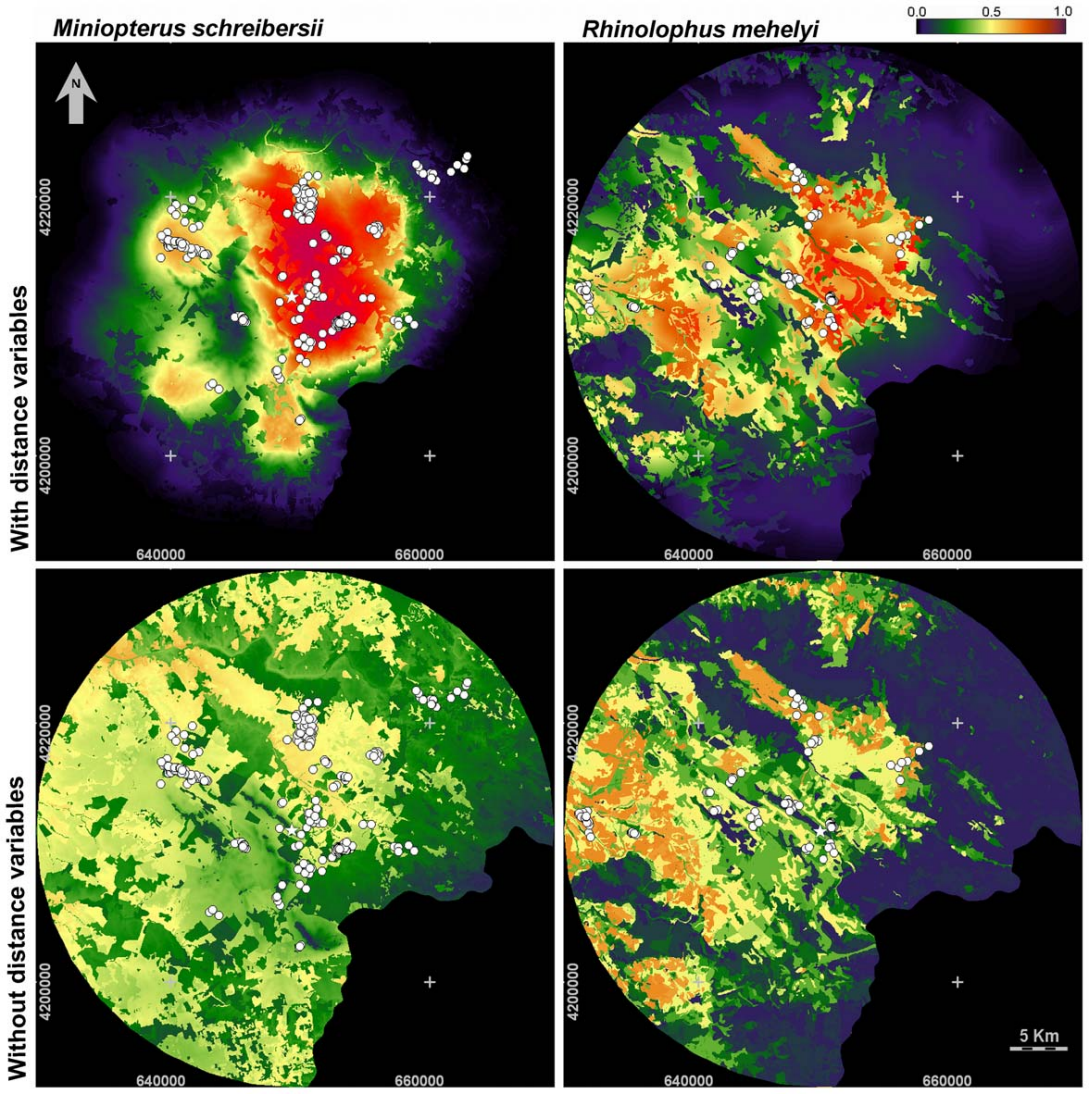
Maps of the predicted foraging suitability of the study area for *Miniopterus schreibersii* (left) and *Rhinolophus mehelyi* (right). These maps were drawn according to the best candidate models including (top) and excluding (bottom) distance variables (see models in Table 2). Habitat suitability is shown on a colour scale ranging from 0 (low suitability) to 1 (high suitability).

The habitat suitability maps created using models with and without distance variables were very different (Figure 4). Areas showed as highly suitable in the latter maps become of low suitability when incorporating distance variables. This is to be expected due to the observed importance of these variables to explain the use of the landscape by the bats.

**Table 2:** Environmental variables used in modelling of the foraging habitat suitability of both bat species.

Variable	Type, units and classes	Source/Scale
Distance to water	Distance to waterlines that maintained water (often in scattered puddles), during the study Continuous, ranging from 0 to 7.5 km.	Derived from IGP [65]
Distance to roost	Continuous, ranging from 0 to 20 km.	Derived from IGP [65]
Distance to urban areas	Distance to towns (>250 inhabitants). Continuous, ranging from 0 to 10 km.	Derived from IGP [65]
Land-cover	Include all main land-cover types of the region. Categorical: Scrub, sparse and dense montado, olive grove, open areas, others.	IGP [65]/1∶25000
Soil capability	A measure of soil productivity, generally for agricultural purposes. Ordinal, ranging from A (high) to E (low productivity).	IDRHa [66]/1∶25000
Altitude	Digital Elevation Model (DEM, SRTM). Continuous, ranging from 80 to 516 m.	Jarvis et al. [67]/90 m
Toposhape	Landscape aspect (see [68]). Categorical: 11 surface shape classes	Derived from SRTM 90 m DEM [67]
NDVI	Normalized Difference Vegetation Index – measure of green biomass. Continuous (−1 to 1)	Derived from Aster 15 m imagery [69]

IGP – Instituto Geográfico Português, IDRHa – Instituto de Desenvolvimento Rural e Hidráulica.

**Figure 5:**
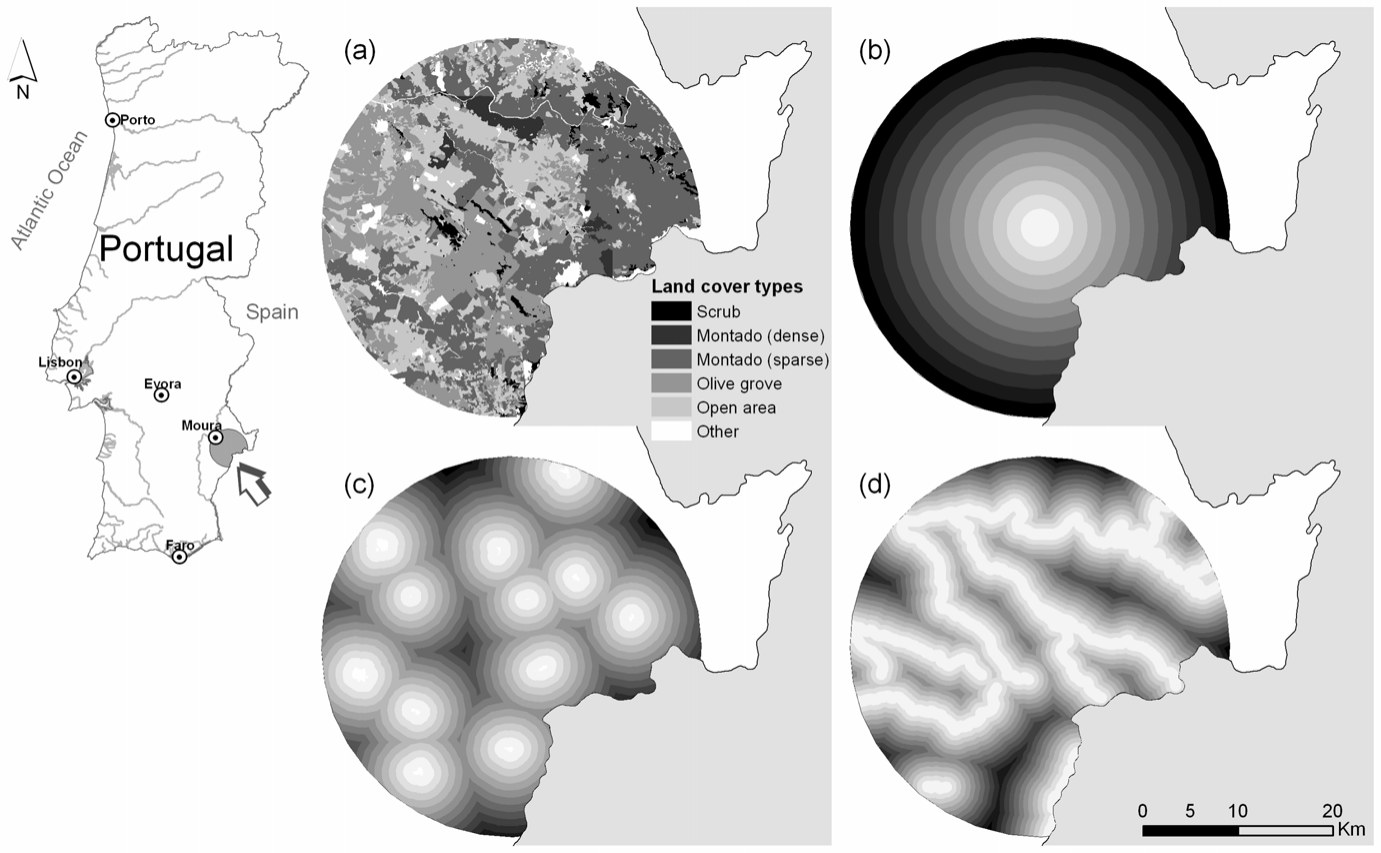
Location of the study area and representation of a subset of the variables analyzed. Variables: land-cover (a), distance to roost (b), distance to urban areas (c) and distance to water (d). Darker shades represent higher distances, in the last three variables.

In the maps built by the models with distance variables (Figure 4) the best foraging areas for *M. schreibersii* are open habitats, near the roost and main waterlines (Figure 5). In the case of *R. mehelyi* the best areas are located near the roost and in a region west of it, which has better soils and two major waterlines. It is also evident that the maps with and without distance variables are far more different in the case of *M. schreibersii* than of *R. mehelyi*. This is a consequence of the higher importance of the distance variables in the models of *M. schreibersii*.

## Discussion

### Importance of distance variables

Although geographic information systems (GIS) environments are well suited to include distance variables as predictors in spatially explicit foraging habitat suitability modelling, this has seldom been done (but see [6], [19], [31]). Such an underuse is somewhat surprising, since it is intuitive that these variables are likely to be of critical importance, particularly in the case of central-place foragers and of highly mobile species that can cover large distances to reach scattered resources, such as many bat and bird species [34].

The results obtained with both *M. schreibersii* and *R. mehelyi* confirmed the importance of using distance variables in the modelling of bat foraging habitat, as the models that included such variables had a much greater explanatory power than those without them. Consequently, the predictive maps of habitat use that we generated with these models also portrayed more accurately how both species use space to forage around their colonies. Various distance variables contributed to the models, and in the remaining of this discussion we will address each of them and the implications of our results for conservation management.

#### Distance to roost

In central-place foragers, individual decisions on the travelling distance from the roost to the foraging sites should result from the compromise between the energy gained by foraging in better locations and lowering competition [34], versus the energy and time lost while commuting [12], [13].

For example, the commuting costs of an individual of the studied bat species, calculated with the models of Speakman and Thomas [15], corresponds approximately to one medium sized moth per kilometre flown. Consequently, to compensate the use of a foraging area located 20 km from the roost, they need about 40 extra moth-captures per night, just to cover the commuting flight. It is evident that the factors that determine the most suitable commuting distance for a central-place foraging bat include both environmental factors, such as the spatial distribution of habitats and the availability of prey, and species specific factors, such as flying costs [13], habitat preferences and the type of prey consumed. Therefore, species are likely to adjust commuting distance differently, as we observed in this study, and this has a dramatic effect on the way they use the landscape for foraging around the colony.

Because commuting distance is such an important factor determining how bats and other central-place foragers use space, models that do not explicitly account for distance to central-place are likely to confound selection with availability [11]. For example, in a particular study area a suitable habitat type may be located so far from the roost that commuting costs make the habitat virtually unavailable for its bats. Without information on distance from roost the modeling results may erroneously indicate that the habitat type is not suitable for foraging.

Conversely, habitat types that are abundant close to the roost may be very used by bats partly because they are highly accessible, and so their suitability may be overrated in the model. Such errors in the understanding of foraging habitat suitability may result in poor management decisions. Likewise, deciding on a habitat management intervention based on a map generated with a model that does not incorporate distance to the colony can be erroneous, as distance may dramatically decrease the potential use of a site, and as we observed this effect may vary from species to species.

#### Distance to water

Riparian areas are often highly profitable foraging grounds for insectivorous bats [35], [36], and rivers are used as orientation landmarks by some species [37]. This may partly explain the presence of distance to water in the models for both species. However, the results show that the positive influence of the presence of water is not limited to its immediate vicinity, and extends for several kilometres (Figure 3). This suggests that the use of riparian habitats for foraging is not the only reason for the importance of this variable. In fact, if bats drink water during their feeding bouts, they may have to forage within an easily reachable source of open water. In our Mediterranean study area summer water sources are rare and unevenly distributed, so distance to water may be a limiting factor in the use of space.

The importance of distance to water to a bat presumably depends on its flight characteristics and on how often it needs to drink while foraging. Little is known about this parameter, but in the one species for which there is data, *Myotis thysanodes*, it varied between 3.7 and 21.2 times per night, in non-lactating and lactating females respectively [18]. In fact, it has been established that bats spend an important proportion of their body water while flying [38].

The results of our models suggest that proximity to a source of drinking water is important for both studied species, and that it does increase habitat suitability. As such, models that incorporate the distance to water variable may be suitable, for example, to plan habitat management interventions in areas that are within adequate reach of water, or to suggest the creation of water points in potentially good foraging habitat that is underused due to the lack of reachable water.

#### Distance to urban areas

Urban development is among the most lasting of the anthropogenic changes of habitat [39], so learning how species react to the presence of urban areas can be important to plan conservation management.

Our results of modelling including the variable distance to urban areas as a potential predictor, demonstrated that this parameter is important for at least some bat species. *M. schreibersii* favoured areas close to urbanizations, and this may be explained by the fact that it often exploits the swarms of insects that concentrate around streetlamps [36], [40], which tend to become increasingly common near urban areas. In contrast, we did not detect any effect of this parameter in the choice of foraging areas by *R. mehelyi*, and this may indicate that they do not forage around street lamps, which would be unusual for a species of *Rhinolophus* [23]. In fact, another species of this genus, *R. hipposideros*, is even known to avoid illuminated areas [24], a possibility that we cannot test for *R. mehelyi*, because our radio-tracking location data do not have sufficient spatial accuracy to determine if it avoided the immediate vicinity of street lamps.

### Relevance for management

Most bat species are colonial, and in many cases their populations are concentrated in a reduced number of large colonies. That is the case for the majority of the bats that form nurseries in underground cavities. All these species are central-place foragers, which tend to be under particular pressure for foraging habitat, because many individuals concentrate their foraging in a relatively small area around the colony roost, as we observed in this study. Consequently, these areas can be of critical importance for an important proportion of the populations of threatened species, and should become a management priority. Bat habitat management can simply consist of the preservation of areas covered with habitats that are highly suitable for foraging, or involve interventions to improve the quality of the habitat. This may include, for example, changing land use [41], applying grazing to control ground vegetation [42], planting hedgerows [43] or promoting organic agriculture [2].

To optimize the use of conservation resources it is important to direct the management interventions to the locations where they have the most potential to maximize conservation effectiveness, and spatially explicit habitat suitability models can help select those locations [3], [44], [45]. Most models used in the past for bats, and for other organisms, use land-cover as a proxy for habitat quality (e.g. [8], [30]). However, it is clear in our results that bat foraging suitability maps based on land-cover alone are very different from those that also incorporate distance variables. Consequently, for both studied species, management decisions based on maps generated with and without distance variables would be very different. For example, many areas that appear to be suitable on the maps generated without distance variables are irrelevant on the maps that include them (Figure 4).

The marked difference between the maps generated for the two species show that distance variables influenced them quite differently. This is not surprising because even closely related species, sharing the same roost, may show distinct foraging requirements and behaviours (e.g. dietary niche breadth, flying ability or energetic requirements [46], [47]). As a consequence, it is important to collect information on the influence of distance for each species, avoiding inter-specific extrapolations that may be incorrect. The influence of distance variables may also vary among regions, so it is preferable that management decisions are based on locally collected data, although this may not always be feasible for logistic reasons.

In conclusion, it is clear that in the case of bats, mostly because of their high mobility and central-place foraging behaviour, distance variables are determinant in the way they use landscapes for foraging around the colony. Distance to roost, to drinking water, and even to urban areas, are among those potentially determinant variables, but others may be equally important, so the selection of the distance factors to include in modelling studies should be based on prior knowledge of the ecology of each species. Incorporating the relevant distance variables in the spatially explicit modelling of bat foraging habitat should result in a clearer interpretation of the importance of the various factors that influence habitat suitability, and in more accurate potential suitability maps. Together, these advantages should improve the guidance provided by modelling in the management of foraging habitats of bats, and of other highly mobile vertebrates that are central-place foragers or that are dependent on spatially scarce resources scattered across landscapes.

## Materials and Methods

### Model species

*Miniopterus schreibersii* (Kuhl, 1817), Schreiber's bent-winged bat, is listed globally as near threatened [48]. It is an agile and efficient flier [49] with long and narrow wings (wingtip index of about 0.88, aspect ratio 6.86 and wing loading 9.13 Nm^−2^), reaches cruising speeds over 50 km/h [50], and may forage in areas as far as 30 km from the roost [40]. It feeds mainly on moths captured in a variety of open, semi-open, natural and artificial habitats, and over watercourses [35], [36], [40].

*Rhinolophus mehelyi* Matschie, 1901, Mehely's horseshoe bat, is listed as vulnerable throughout its range [51]. It feeds mainly on moths [52], but its foraging behaviour is poorly known. A study performed in southern Spain indicates that this species forages in relatively open habitats [46]. Its wing morphology is quite different from that of *M. schreibersii*, with a lower aspect ratio (5.81), and rounded wingtips (wingtip index 1.71) suggesting that, like most Rhinolophids, it is able to forage close to vegetation in highly structured habitats. However, its wing loading (8.48 Nm^−2^) indicates that it is also capable of fast commuting flight [53].

Both species are included in Annexes II and IV of the 92/43/EEC European Union Council Directive, which requires them to be the focus of specific measures designed to maintain or restore their favourable conservation status.

### Study area

The studied colonies of *R. mehelyi* and *M. schreibersii* roost in the same abandoned mine in south-eastern Portugal (38°02′N 7°17′E; Figure 5). This is a dry region, characterized by a Mediterranean-Continental climate with marked seasonal variations in rainfall and temperature.

It is mostly flat, with gentle slopes (200–500 m asl) and poor soils. It is sparsely populated (17 inhabitants per km^2^) and most people live in small villages. The dominant land use is a silvo-pastoral system called montado, which is mostly on poor soils, and usually consists of vast grasslands with a tree cover of holm oak (*Quercus rotundifolia*) or cork oak (*Quercus suber*). The livestock used in this extensive system is primarily cattle, but sheep and Iberian black pigs are also common. The second most important land use in the region are olive groves (*Olea europaea*), which consist of rows of trees on sparsely covered ground and are located on a broad range of soils. Cereal crops (wheat and barley) and fodder, mostly planted on near treeless fields, tend to occupy the best soils. The water lines are usually narrow and shallow, and characterized by a highly variable intra-annual flow. The use of these habitats by bats has been described elsewhere [35].

Part of this area is included in the Natura 2000 network Moura-Barrancos site (under European Union Council Directive 79/409/EEC; http://ec.europa.eu/environment/nature/index_en.htm). Despite its recognized natural value the area is under pressure from agriculture intensification, particularly with irrigated crops and high density olive plantations.

### Foraging behaviour

Foraging behaviour of both species was studied by radio-tracking. Bats were captured inside the roost between May and July during six consecutive years. In Portugal the authority responsible for issuing all the permits for capturing, handling, and working with wild animals is the *Instituto para a Conservação da Natureza e da Biodiversidade*. We worked with yearly permits numbers: 18/1997/Capt, 12/1998/Capt, 15/1999/Capt, 10/2000/Capt and 12/2002/Capt.

Each individual was weighed and ringed, and a small transmitter (BD-2A, 0.47 g; Holohil Systems, ON, Canada) was glued between its shoulder blades using Skinbond adhesive (Smith-Nephew United, Largo, FL, USA). The weight of the transmitter was <5% of that of the animal, to avoid affecting the activity of the tracked bat [54]. After attaching the transmitter, bats were released inside the roost.

Five *M. schreibersii* and four *R. mehelyi* were captured to obtain wing parameters. We measured these on pictures of right wings fully extended over graph paper. Wing loading, aspect ratio and wingtip index were calculated following Norberg and Rayner [55].

Bats were tracked using a network of three to five fixed telemetry stations located on high strategic points of the study area, and one mobile tower mounted on a four-wheel drive vehicle. Each fixed telemetry station consisted of an 8-m high metal tower supporting two parallel 6-element Yagi antennas, connected to a precision null combiner (Tac-5, Telonics, Mesa, AZ, USA) and to a telemetry receiver (TRX-1000S, Wildlife Materials, Carbondale, IL, USA). The mobile station was similar, but had 3-element Yagi antennas positioned 6 m above the ground. Each receiving station was calibrated daily and the observed error was generally below 2°. The operators of the telemetry stations were in permanent radio contact to allow the reading of simultaneous bearings every 5 minutes, whenever the tracked bats were within the range of the antennas.

### Analysis of radio-tracking data

Bat locations were calculated by triangulation from the radio-tracking data, and were then screened using the techniques described by White and Garrot [56] to eliminate potentially inaccurate results. We also excluded all locations of commuting bats (speeds usually above 4.7 m/s for both species), keeping only those where the slower movements and permanence in an area suggested that the animals were foraging.

Most methods to analyse the use of space by tracked animals assume that consecutive locations of the same animal are independent, which is often not the case (see [57] for a review). To minimize the problems caused by this potential spatio-temporal autocorrelation, we used the following approach adapted from Boyce et al. [58]. A Moran's I correlogram was built for each environmental variable, using random points located from 0 to 20 km apart, with GStat in Idrisi (v.14.02 Kilimanjaro, Clark University, Worcester, MA, USA). To test for significant autocorrelations, we calculated Z-score values for each lag distance, using ArcInfo 9.0 (ESRI, Redlands, CA, USA), and concluded that none of the variables had significant autocorrelation at distances over four kilometres. Since we observed that it took both species about 11 minutes to fly this distance, we used this interval as the time to independence between successive locations.

### Habitat suitability modelling

We investigated the potential role of several landscape and distance variables as predictors of the presence of foraging bats (Table 2). After a graphical analysis to visually explore the relationship between each predictor and the presence/absence of bats, we tested it using univariate logistic regressions [59]. For absences we used a set of locations randomly distributed throughout the study area [11], but excluding those that fell close (<500 m) to sites where bats were observed [60]. This procedure enables the inclusion of absences distributed in the area of potential foraging, but outside the immediate environmental domain of the presences, and has been recommended to reduce the number of false absences [61]. We used 124 and 356 foraging locations for *R. mehelyi* and *M. schreibersii* respectively, and an equal number of absences. Variables with a regression p-level over 0.3 – Toposhape and NDVI in both species (Table 2) – were excluded from further analyses [62], because there was no additional biological evidence that justified their inclusion. A Spearman correlation matrix was generated to check for collinearity between the remaining variables, but all correlation values were below 0.7 [63].

We then generated two sets of candidate habitat suitability models for each species: (i) considering all possible combinations of the selected variables and plausible interactions, and (ii) excluding the three distance variables from the initial dataset of selected variables. For each set and for each species, the model with the smallest Akaike's Information Criterion corrected for small sample size (AICcmin; Burnham and Anderson 2002) was considered the best candidate. The difference between this AICcmin and the AICc values of the remaining models (Δi) and the analysis of receiver-operating characteristics (ROC, [64]), were used to determine the performance of each model. Model selection was done with the package Multi-model Inference (MuMIn) in the R environment (v. 2.10.1, The R Foundation for Statistical Computing, Vienna, Austria). Finally, the best candidate models were used to generate foraging suitability maps with a GIS.

## Supporting Information

Table S1 Individual data and tracking survey data of followed *Rhinolophus mehelyi* and *Miniopterus schreibersii*.
(PDF)
