# Supplementary material for: The Importance of Distance to Resources in the Spatial Modelling of Bat Foraging Habitat
Source: PLoS One. 2011 Apr 25;6(4):e19227. doi: 10.1371/journal.pone.0019227 (PMC3081845; doi:10.1371/journal.pone.0019227)
Supplement: Table S1 — Individual data and tracking survey data of followed Rhinolophus mehelyi and Miniopterus schreibersii. (PDF) [file pone.0019227.s001.pdf]

Table S1 Individual data and tracking survey data of followed *Rhinolophus mehelyi* and *Miniopterus schreibersii*.

Year	Capture	Species	Sex	Tracking nights	Tracking hours	Fixes	Fixes while foraging
1997	20-05	*R. mehelyi*	F	7	8:00	32	10
	21-05	*R. mehelyi*	F	10	8:25	33	9
	13-06	*R. mehelyi*	F	5	7:55	31	7
1998	22-05	*R. mehelyi*	F	5	9:30	38	20
	28-05	*R. mehelyi*	F	4	6:00	24	8
	30-05	*M. schreibersii*	F	8	28:50	115	32
	30-05	*R. mehelyi*	F	5	4:30	18	0
	03-06	*M. schreibersii*	F	3	9:30	38	13
	15-06	*M. schreibersii*	F	5	18:50	75	40
	15-06	*R. mehelyi*	F	6	8:00	32	0
	25-06	*M. schreibersii*	F	9	53:55	215	97
	25-06	*R. mehelyi*	F	3	16:00	64	35
	01-07	*R. mehelyi*	F	2	4:25	17	7
1999	27-06	*M. schreibersii*	F	4	14:25	57	21
	29-06	*M. schreibersii*	F	9	54:30	218	55
	19-07	*M. schreibersii*	F	5	4:30	18	0
	24-07	*M. schreibersii*	F	5	53:30	214	132
2000	17-05	*M. schreibersii*	F	6	1:25	32	0
	25-05	*M. schreibersii*	F	3	4:00	16	0
	11-06	*M. schreibersii*	F	2	3:75	15	0
	19-06	*M. schreibersii*	F	5	43:25	173	138
	21-06	*M. schreibersii*	F	3	9:00	36	14
2002	15-06	*R. mehelyi*	F	5	8:00	96	77
	30-06	*R. mehelyi*	F	3	5:30	22	15
